# Attention Contributes to Arithmetic Deficits in New-Onset Childhood Absence Epilepsy

**DOI:** 10.3389/fpsyt.2017.00166

**Published:** 2017-09-14

**Authors:** Dazhi Cheng, Xiuxian Yan, Zhijie Gao, Keming Xu, Qian Chen

**Affiliations:** ^1^Department of Pediatric Neurology, Capital Institute of Pediatrics, Beijing, China

**Keywords:** childhood absence epilepsy, attention, executive function, arithmetic performance, neuropsychological test

## Abstract

Neuropsychological studies indicate that new-onset childhood absence epilepsy (CAE) is associated with deficits in attention and executive functioning. However, the contribution of these deficits to impaired academic performance remains unclear. We aimed to examine whether attention and executive functioning deficits account for the academic difficulties prevalent in patients with new-onset CAE. We analyzed cognitive performance in several domains, including language, mathematics, psychomotor speed, spatial ability, memory, general intelligence, attention, and executive functioning, in 35 children with new-onset CAE and 33 control participants. Patients with new-onset CAE exhibited deficits in mathematics, general intelligence, attention, and executive functioning. Furthermore, attention deficits, as measured by a visual tracing task, accounted for impaired arithmetic performance in the new-onset CAE group. Therefore, attention deficits, rather than impaired general intelligence or executive functioning, may be responsible for arithmetic performance deficits in patients with new-onset CAE.

## Introduction

Childhood absence epilepsy (CAE) is a common type of epilepsy that affects approximately 8% of preschoolers and school children with epilepsy (1). CAE is clinically characterized by a typical electroencephalography (EEG) pattern, frequent staring spells, and generalized 3-Hz spike-wave discharges (2). Although CAE was previously considered to be a “benign” epilepsy syndrome that is relatively easy to control (3, 4), more recent studies have reported that children with CAE exhibit neurocognitive functional deficits (1, 5, 6). In particular, patients with CAE demonstrate deficits in visuospatial skills (1), attention (6), and verbal memory (7), and executive functioning and motor control (8). Attention and executive functioning deficits have been reported even in patients without seizures (5). In addition, impairments in attention, executive functioning, and academic performance (including language and mathematics) have been reported even in patients who have been newly diagnosed with CAE (5, 9).

Impairments in executive functioning negatively impact academic performance and achievement (5). However, the relationships between attention or executive functioning and academic performance in patients with new-onset CAE have not been elucidated. In this study, we evaluated a group of children with newly diagnosed and untreated CAE, to test the hypothesis that patients with new-onset CAE would exhibit deficits in attention and executive functioning that may account for academic difficulties.

## Materials and Methods

### Patients and Control Group Selection

**Figure 1:**
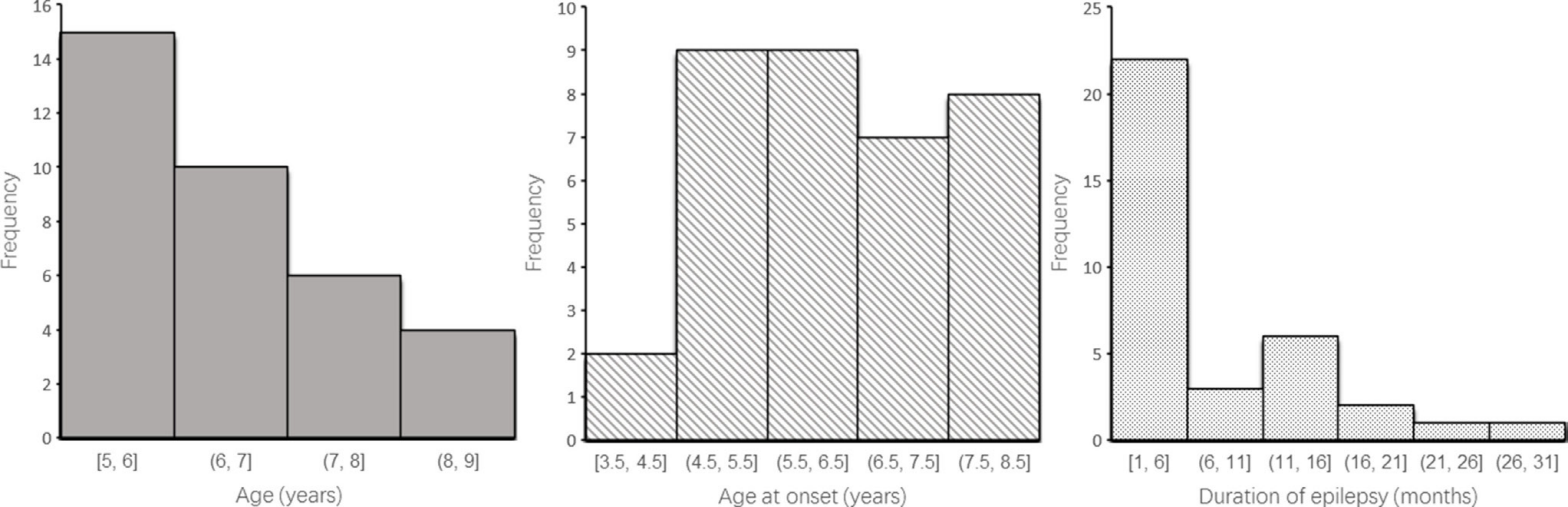
Histogram of patients’ characterization such as age, age at onset, and time to diagnosis.

This study recruited 35 patients (14 boys and 21 girls) with a history of untreated typical CAE, from the Department of Pediatric Neurology at the Capital Institute of Pediatrics in Beijing of China (Table 1; Figure 1). Enrolled patients met the following inclusion criteria: (1) clinical diagnosis of CAE according to the classifications of epileptic syndromes of the International League Against Epilepsy (10); (2) EEG showing a normal background and 3–4 Hz generalized spike and wave discharges during the ictal period, or an absence attack induced by hyperventilation; and (3) being newly diagnosed and drug naive. The exclusion criteria were as follows: (1) presence of any other type of seizure or seizure disorder, including tonic-clonic, myoclonic, and partial seizures; (2) any previous exposure to drugs; (3) any brain abnormality evident in magnetic resonance imaging or any other neurological disease; and (4) inability to independently complete the tasks. The treatment was initiated in 1 day immediately after the neuropsychological testing.

**Table 1:** Demographic characteristics of patients with new-onset CAE and control participants (mean ± SD).

Characteristics	New-onset CAE (*n* = 35)	Control (*n* = 33)	*P* value
Age (years), mean ± SD (range)	7.3 ± 1.3 (5–10)	6.8 ± 1.1 (5–9)	0.14
Gender (boys/girls)	14/21	18/15	0.23
Age at onset, years (SD)	6.7 (1.3)	N/A	N/A
Duration of epilepsy (time to diagnosis), months (SD)	7 (7)	N/A	N/A

*N/A, not applicable; CAE, childhood absence epilepsy*.

The control group included 33 healthy participants (18 boys and 15 girls), who were recruited from a local primary school and a junior high school in Beijing. The mean age at the time of testing was 7.5 years (range, 5–12 years). The control group was matched with the CAE group for age and sex. This study was approved by the Human Research Ethics Committee at the Capital Institute of Pediatrics (approval no.: SHERLL 2015023). Written informed consent was obtained from the parents of all participants prior to research participation.

### Neuropsychological Tests

Participants in the patient and control groups completed a neuropsychological test battery that included measures of general cognitive processing (i.e., psychomotor speed, spatial ability, memory, general intelligence, attention, and executive function), language processing (i.e., semantic comprehension), and mathematical processing (i.e., arithmetic and numerical magnitude processing). Table 2 provides details regarding the target cognitive domains and the specific abilities assessed by each test.

**Table 2:** Neuropsychological test battery.

Domain	Ability	Tests
Language	Semantic comprehension	Word semantics
Mathematics	Numerical magnitude processing	Number comparison
	Arithmetic/computational fluency	Simple subtraction
Psychomotor speed	Processing speed	Choice reaction time
Spatial ability	Spatial perception	Mental rotation
Memory	Forward verbal working memory	Forward digit span
	Backward verbal working memory	Backward digit span
General intelligence	Non-verbal matrix reasoning	Raven’s Progressive Matrices
Attention	Visual attention	Visual tracing
Executive functioning	Response inhibition and mental flexibility	Wisconsin Card Sorting Test

All tests were programmed using web-based applications from the Online Experimental Psychological System (www.dweipsy.com/lattice) (11). For all, but two, tasks children responded by pressing one of two keys (“P” or “Q”) on a computer keyboard; for visual tracing and the Wisconsin Card Sorting Test, participants clicked a mouse to choose the correct answer. Participant responses were automatically recorded by the computer and relayed to a server *via* the Internet.

#### Word Semantics

This task was similar to a previously reported task that evaluated semantic comprehension of language ability (12, 13). Task stimuli were selected from textbooks used in primary schools between the first and ninth grades. For each trial, a sentence containing a missing word was presented in the middle of a computer screen, and two candidate words were presented beneath the sentence. To complete the sentence, participants pressed the “Q” key to choose the answer on the left and the “P” key to choose the answer on the right.

#### Number Comparison Task

This task was adapted from a previously described number comparison test to evaluate numerical magnitude processing (14). One-digit Arabic numbers of identical or differing size were presented on the computer screen. Participants were asked to decide which number had a larger numerical magnitude, irrespective of the physical size of the numbers. The ratio of differently sized numbers was 1:2. Participants pressed the “Q” or “P” key to choose the answer. The task consisted of 84 trials separated into 3 sessions (i.e., 28 trials per session) in a randomized order, with two 30-s rest periods.

#### Simple Subtraction

This task consisted of 92 simple subtraction problems (e.g., 6 − 2, 17 − 8). The minuends were 18 or smaller, and the differences were single-digit numbers (11). Two candidate answers were presented beneath each problem. Participants pressed the “Q” or “P” key to choose the answer. Each incorrect candidate answer was within 1–3 values of the correct answer (i.e., ±1, ±2, or ±3). This task was time limited to 2 min and usually evaluated arithmetic or computational fluency ability (15).

#### Choice Reaction Time

This task was adapted from a previously described task that was used to evaluate processing speed (16). For each trial, a white dot was presented to the right or to the left of a fixation cross on a black screen. The localization of the dot was within 15° of the visual angle of the cross. Participants pressed the “Q” or “P” key to judge the localization of the white dot. The task consisted of 30 randomized trials with the duration of interstimulus interval randomly varying between 1,500 and 3,000 ms.

#### Mental Rotation

This task was adapted from the mental rotation task to assess spatial perception (17). For each trial, one three-dimensional figure (i.e., the target figure) was presented on the top of the screen, and two candidate figures were presented beneath the target figure. Participants had to select the figure that represented a rotated version of the target figure by pressing the “Q” or “P” key. The matching figure varied between a 15° and 345° rotation angle, with an interval of 15°, whereas each non-matching figure was the rotated mirror image of the target figure. The test consisted of 180 trials in total and was time limited to 3 min.

#### Forward or Backward Digit Spans

This task was adapted from the digit span test of the Wechsler Intelligence Scale, which tests verbal working memory (18). A series of digits was presented orally through a pair of earphones, and each lasted for 200 ms. The test began with two items (digits) for forward digit spans and increased gradually until the participant failed to input the digits three consecutive times. For forward digit spans, participants needed to input the digits in accordance with the digit order, whereas for backward digit spans, participants were asked to type the digits according to the reverse order of the digits. The test lasted approximately 10 min, but was not time limited.

#### Raven’s Progressive Matrices

A simplified version of Raven’s Progressive Matrices was used to evaluate general intelligence (19). Participants were asked to identify the missing segment that would complete a figure’s pattern. Participants pressed the “Q” or “P” key to choose the candidate answers beneath each problem. The test consisted of 80 trials and was time limited to 3 min. The task was used to evaluate non-verbal matrix reasoning.

#### Visual Tracing

This assessment was adapted from a previously described visual tracing test to evaluate visual attention (20). Several curved lines were interwoven within a square, starting from the left side and ending on the right side. Participants were asked to track a specified line using only their eyes and to mark the correct end point. The degree of difficulty increased as the number of lines increased. The task consisted of 12 trials, each with 3 target lines, and duration was 4 min.

#### Wisconsin Card Sorting Test

This task was adapted from the manual version of the Wisconsin Card Sorting Test, which is used to assess response inhibition and mental flexibility of executive functioning (21, 22). The task stimuli included target cards and response cards. Participants were asked to turn over response cards by clicking a mouse and to match each response card with one of the target cards according to one of three principles: color, form, or quantity. Participants were asked to determine the sorting principle based on feedback (a smiling face indicating a correct response or a sad face indicating an incorrect response). Duration of this task was 20 min.

### Procedure

The neuropsychological test battery was administered to participants in two 45-min sessions, in an examination room. For each test, instructions were given, followed by a practice session. The practice session contained either four or six trials, similar to the formal test session. Participants were free to ask questions to test administrators during the practice session. After the practice session, participants could press any key to begin the formal test.

The tests were conducted in the same order for all participants. Each participant was monitored by one tester who was familiar with the standardized testing procedures. All data were collected from January 2014 to March 2016.

### Statistical Analyses

For all, but one, tests (i.e., the choice reaction time test), corrected scores were calculated by subtracting the number of incorrect responses from the number of correct responses to account for guessing (14, 23–25). For the choice reaction time test, the median reaction time was calculated. One-way analysis of variance (ANOVA) compared performance (i.e., continuous variables) on all tests between the new-onset CAE group and the control group. Chi-squared tests were used to analyze sex differences (i.e., categorical variables).

Analysis of covariance (ANCOVA) was conducted to examine the relationships among general cognitive processing, language processing, and mathematical abilities. Attention and executive functioning, as well as other cognitive measures (i.e., choice reaction time, mental rotation, forward and backward digit span, and Raven’s Progressive Matrices), were also treated as covariates to test mathematical processing differences between the groups. In addition, correlation analyses were performed to investigate the relationships between key study measures.

## Results

### Population Characteristics

The demographic and neurological characteristics of participants are summarized in Table 1. No differences were found between the new-onset CAE group and the control group with respect to age (*F* = 0.19, *p* = 0.66) or sex (χ^2^ = 1.44, *p* = 0.23).

### Neurocognitive Profiles

The mean score and SD for all tests, according to group, are listed in Table 3. The one-way ANOVA showed that the new-onset CAE group had impaired performance compared to the control group on three tests of general cognitive processing: *F*_1, 66_ = 5.71, *p* < 0.05, and $\eta_{\text{p}}^{2}$ = 0.080 for Raven’s Progressive Matrices;*F*_1, 66_ = 7.60, *p* < 0.01, and $\eta_{\text{p}}^{2}$ = 0.103 for visual tracing; and*F*_1, 66_ = 12.36, *p* < 0.001, and $\eta_{\text{p}}^{2}$ = 0.158 for the Wisconsin Card Sorting test. The new-onset CAE group also had impaired performance compared to the control group on the two arithmetic performance tests: *F*_1, 66_ = 4.87, *p* < 0.05, $\eta_{\text{p}}^{2}$ = 0.069 for number magnitude comparison; and *F*_1, 66_ = 8.15, *p* < 0.01, $\eta_{\text{p}}^{2}$ = 0.110 for simple subtraction. For word semantics, there was no significant difference between the new-onset CAE and the control group (*F*_1, 66_ = 1.18, *p* = 0.28). There were also no significant between-group differences for choice reaction time, mental rotation, or forward and backward digit span tasks.

**Table 3:** Mean (SD) values and one-way ANOVAs of cognitive test scores in patients with new-onset CAE (*n* = 35) and control participants (*n* = 33).

Tests	New-onset CAE	Control	*F*
Word semantics	9.34 (9.44)	12.15 (11.79)	1.18
Number comparison	54.74 (29.74)	66.85 (10.69)	4.87*
Simple subtraction	23.26 (10.37)	30.67 (11.04)	8.15**
Choice reaction time	619.40 (174.41)	547.58 (250.28)	1.90
Mental rotation	10.14 (9.31)	12.73 (10.44)	1.16
Forward digit span	3.80 (3.33)	4.88 (3.40)	1.75
Backward digit span	2.54 (2.43)	3.33 (2.06)	2.09
Raven’s Progressive Matrices	10.94 (7.96)	15.27 (6.92)	5.71*
Visual tracing	6.11 (5.64)	9.76 (5.23)	7.60**
Wisconsin Card Sorting Test	45 (29.15)	65.97 (18.54)	12.36**

*p < 0.05.

**p < 0.01.

*CAE, childhood absence epilepsy; ANOVA, analysis of variance*.

*All children in both groups completed all tests*.

The ANCOVA demonstrated that after controlling for the four cognitive ability test scores (i.e., choice reaction time, mental rotation, forward and backward digit span, and Raven’s Progressive Matrices), between-group differences for number comparisons were not observed (*F*_1, 61_ = 2.33, *p* = 0.13), whereas the group difference for simple subtraction remained significant (*F*_1, 61_ = 4.15, *p* < 0.05, and $\eta_{\text{p}}^{2}$ = 0.064). With additional control for executive functioning, the group difference for simple subtraction, still, marginally attained significance (*F*_1, 60_ = 3.72, *p* = 0.058, and $\eta_{\text{p}}^{2}$ = 0.058). However, after additional control for attention, the between-group difference for simple subtraction disappeared (*F*_1, 59_ = 1.31, *p* = 0.26).

Correlations between task scores are displayed in Table 4. Number comparison scores significantly correlated with mental rotation, forward digit span, backward digit span, and Raven’s Progressive Matrices scores. The simple subtraction scores were also significantly correlated with choice reaction, mental rotation, Raven’s Progressive Matrices, and visual tracing scores.

**Table 4:** Correlations between test scores of all participants.

	1	2	3	4	5	6	7	8	9
1. Word semantics	–								
2. Number comparison	0.23	–							
3. Simple subtraction	0.60**	0.28*	–						
4. Choice reaction time	−0.34**	−0.06	−0.46**	–					
5. Mental rotation	0.37**	0.34**	0.34**	−0.32**	–				
6. Forward digit span	0.16	0.30*	0.10	0.05	0.10	–			
7. Backward digit span	0.09	0.34**	0.23	−0.19	0.13	0.58**	–		
8. Raven’s Progressive Matrices	0.31*	0.27*	0.31**	−0.33**	0.50**	0.23	0.24*	–	
9. Visual tracing	0.30*	0.13	0.58**	−0.42**	0.28*	0.24*	0.54**	0.30*	–
10. Wisconsin Card Sorting Test	0.06	0.05	0.16	−0.04	0.15	0.30*	0.34**	0.21	0.19

*p < 0.05.

**p < 0.01.

## Discussion

This study examined neurocognitive deficits in patients with new-onset CAE to clarify the contributions of attention and executive functioning in academic performance and achievement. The results demonstrated that children with new-onset CAE have cognitive deficits in general intelligence, attention, executive functioning, and mathematical abilities. We further analyzed the relationships between attention and executive functioning and impaired arithmetic performance. Attention deficits, measured by the visual tracing test, accounted for arithmetic performance in the new-onset CAE group. These results suggested that attention may be responsible for arithmetic performance deficits in patients with new-onset CAE.

The current investigation first replicated previous findings that neurocognitive deficits in attention and executive functioning occur in patients with new-onset CAE (5). Although a previous study showed general intelligence and arithmetic performance deficits in patients with new/recent-onset absence epilepsy, the sample of children with absence epilepsy in this previous study was small (*n* = 11) (9). In the larger sample presented here, children with new-onset CAE had significantly decreased cognitive performance on measures of general intelligence and arithmetic performance, relative to healthy control participants. In contrast, deficits were not detected in processing speed, spatial perception, memory, or language processing. In addition to attention and executive functioning deficits, general intelligence and arithmetic performance deficits should be regarded as cognitive characteristics of new-onset CAE.

Our results demonstrated that patients with new-onset CAE exhibited arithmetic deficits rather than deficits in language ability. Patients with CAE have network impairments involving the frontal operculum and medial frontal cortex (26, 27), which include brain regions involved in arithmetic processing in the medial frontal lobe (28). In contrast, processing of semantic knowledge for language is thought to occur in the left inferior prefrontal cortex (29). Therefore, our finding that patients with new-onset CAE only had cognitive deficits in mathematical academic achievement is consistent with previous findings.

In this study, attention, rather than general intelligence or executive functioning, accounted for arithmetic deficits in patients with new-onset CAE. In contrast, Masur et al. found that attention did not directly affect academic achievement, but had secondarily influenced achievement scores through memory (5); this finding may have been due to the use of a composite academic achievement score that included assessments of both reading and arithmetic skills. Consistent with the present findings, previous studies have demonstrated that attention is strongly associated with arithmetic performance; for example, 42% of children with dyscalculia also experience attention deficits (30). Moreover, sustained visual attention ability can predict mathematical but not reading performance (31).

Study limitations include a relatively small sample size, statistical approach, and incomplete mathematical assessment. New-onset CAE patients with CAE are rare in our hospital, and it is difficult to secure large enrollment. Our findings will require confirmation in a larger sample of children with new-onset CAE. ANCOVA was conducted to examine the relationships among cognitive abilities and arithmetic performance. In comparison, structural equation modeling can be used to infer the relationships and causation. If a larger sample is enrolled, it may be possible to use structural equation modeling (5) to interpret the interaction effect between neuropsychological factors and academic achievement. However, by using a common assessment approach of computational fluency as a measure of arithmetic performance (15), we confirmed the study’s hypotheses that impaired arithmetic performance occurs in patients with new-onset CAE and that attention deficits contribute to arithmetic performance in these patients.

## Conclusion

This study extends previous neuropsychological findings by confirming neurocognitive deficits in children with new-onset CAE and also illustrates that impairments in attention, rather than in other cognitive functions, account for arithmetic performance in these patients. We conclude that attention deficits may be involved in arithmetic performance deficits in patients with new-onset CAE comparing with healthy controls. Future neuropsychological assessment studies using larger cohorts are required to validate our findings and further improve the understanding of newly diagnosed CAE.

## Ethics Statement

This study was approved by the Human Research Ethics Committee at the Capital Institute of Pediatrics (approval no.: SHERLL 2015023).

## Author Contributions

DC and QC conceived and designed the experiment. DC, XY, and ZG performed the experiment. DC and XY analyzed the data. DC, XY, ZG, KX, and QC wrote the paper. DC, XY, QX, XZ, and QC revised the manuscript.

## Conflict of Interest Statement

The authors declare that the research was conducted in the absence of any commercial or financial relationships that could be construed as a potential conflict of interest.

## References

[1] Pavone P Bianchini R Trifiletti RR Incorpora G Pavone A Parano E. Neuropsychological assessment in children with absence epilepsy. Neurology (2001) 56:1047–51. 10.1212/WNL.56.8.1047 11320177

[2] Loiseau P Duche B Pedespan JM. Absence epilepsies. Epilepsia (1995) 36:1182–6. 10.1111/j.1528-1157.1995.tb01060.x 7489694

[3] Covanis A Skiadas K Loli N Lada C Theodorou V Absence epilepsy: early prognostic signs. Seizure (1992) 1:281–9. 10.1016/1059-1311(92)90038-3 1344778

[4] Dieterich E Doose H Baier WK Fichsel H. Longterm follow-up of childhood epilepsy with absences. II. Absence-epilepsy with initial grand mal. Neuropediatrics (1985) 16:155–8. 10.1055/s-2008-1059531 3930989

[5] Masur D Shinnar S Cnaan A Shinnar RC Clark P Wang J Pretreatment cognitive deficits and treatment effects on attention in childhood absence epilepsy. Neurology (2013) 81:1572–80. 10.1212/WNL.0b013e3182a9f3ca 24089388 PMC3806916

[6] Vega C Vestal M DeSalvo M Berman R Chung M Blumenfeld H Differentiation of attention-related problems in childhood absence epilepsy. Epilepsy Behav (2010) 19:82–5. 10.1016/j.yebeh.2010.06.010 20674507 PMC2943027

[7] Nolan MA Redoblado MA Lah S Sabaz M Lawson JA Cunningham AM Memory function in childhood epilepsy syndromes. J Paediatr Child Health (2004) 40:20–7. 10.1111/j.1440-1754.2004.00284.x 14717999

[8] Conant LL Wilfong A Inglese C Schwarte A. Dysfunction of executive and related processes in childhood absence epilepsy. Epilepsy Behav (2010) 18:414–23. 10.1016/j.yebeh.2010.05.010 20656561

[9] Jackson DC Dabbs K Walker NM Jones JE Hsu DA Stafstrom CE The neuropsychological and academic substrate of new/recent-onset epilepsies. J Pediatr (2013) 162:1047–53.e1. 10.1016/j.jpeds.2012.10.046 23219245 PMC3615134

[10] Engel J A proposed diagnostic scheme for people with epileptic seizures and with epilepsy: report of the ILAE Task Force on Classification and Terminology. Epilepsia (2001) 42:796–803. 10.1046/j.1528-1157.2001.10401.x 11422340

[11] Wei W Lu H Zhao H Chen C Dong Q Zhou X. Gender differences in children’s arithmetic performance are accounted for by gender differences in language abilities. Psychol Sci (2012) 23:320–30. 10.1177/0956797611427168 22344448

[12] Mummery CJ Patterson K Hodges JR Price CJ. Functional neuroanatomy of the semantic system: divisible by what? J Cogn Neurosci (1998) 10:766–77. 10.1162/089892998563059 9831743

[13] So D Siegel LS Learning to read Chinese: semantic, syntactic, phonological and working memory skills in normally achieving and poor Chinese readers. Read Writ (1997) 9:1–21. 10.1023/A:1007963513853

[14] Hedden T Yoon C. Individual differences in executive processing predict susceptibility to interference in verbal working memory. Neuropsychology (2006) 20:511–28. 10.1037/0894-4105.20.5.511.Supp 16938014

[15] Zhou X Wei W Zhang Y Cui J Chen C. Visual perception can account for the close relation between numerosity processing and computational fluency. Front Psychol (2015) 6:1364. 10.3389/fpsyg.2015.01364 26441740 PMC4563146

[16] Butterworth B Dyscalculia Screener. London: NFER-Nelson (Software and Manual) (2003).

[17] Vandenberg SG AR K Mental rotations, a group test of three-dimensional spatial visualization. Percept Motor Skills (1978) 47:599–604. 10.2466/pms.1978.47.2.599 724398

[18] Wechsler D Manual for the Wechsler Intelligence Scale for Children, Revised. San Antonio, TX: Psychological Corp. (1974).

[19] Raven J Jourt J Raven’s Progressive Matrices and Vocabulary Scales. Oxford, UK: Oxford Psychologists Press (1998).

[20] Groffman S Visual tracing. J Am Optom Assoc (1966) 37:139–41.

[21] Heaton RK Wisconsin Card Sorting Test Manual, Psychological Assessment Resources. Odessa, FL: Psychological Assessment (1993).

[22] Grant DA Berg EA A behavioral analysis of degree of reinforcement and ease of shifting to new responses in a Weigl-type card-sorting problem. J Exp Psychol (1948) 38:404–11. 10.1037/h0059831 18874598

[23] Cirino PT. The interrelationships of mathematical precursors in kindergarten. J Exp Psychol (2011) 108:713–33. 10.1016/j.jecp.2010.11.004 21194711 PMC3043138

[24] Salthouse TA The nature of the influence of speed on adult age differences in cognition. Dev Psychol (1994) 30:240–59. 10.1037/0012-1649.30.2.240 7983473

[25] Salthouse TA Meinz EJ. Aging, inhibition, working memory, and speed. J Gerontol B Psychol Sci Soc Sci (1995) 50:297–306. 10.1093/geronb/50B.6.P297 7583809

[26] Bai X Guo J Killory B Vestal M Berman R Negishi M Resting functional connectivity between the hemispheres in childhood absence epilepsy. Neurology (2011) 76:1960–7. 10.1212/WNL.0b013e31821e54de 21646622 PMC3109878

[27] Carney PW Masterton RA Flanagan D Berkovic SF Jackson GD. The frontal lobe in absence epilepsy: EEG-fMRI findings. Neurology (2012) 78:1157–65. 10.1212/WNL.0b013e31824f801d 22459682

[28] Simon O Mangin JF Cohen L Bihan DL Dehaene S. Topographical layout of hand, eye, calculation, and language-related areas in the human parietal lobe. Neuron (2002) 33:475–87. 10.1016/S0896-6273(02)00575-5 11832233

[29] Thompson-Schill SL Farah MJ. Role of left inferior prefrontal cortex in retrieval of semantic knowledge: a reevaluation. Proc Natl Acad Sci U S A (1997) 94:14792–7. 10.1073/pnas.94.26.14792 9405692 PMC25116

[30] Lindsay RL Tomazic T Levine MD Accardo PJ. Attentional function as measured by a continuous performance task in children with dyscalculia. J Dev Behav Pediatr (2001) 22:287–92. 10.1097/00004703-200110000-00002 11718231

[31] Anobile G Stievano P Burr DC. Visual sustained attention and numerosity sensitivity correlate with math achievement in children. J Exp Child Psychol (2013) 116:380–91. 10.1016/j.jecp.2013.06.006 23933254

